# A case report of ultrasonographic findings in bilateral diffuse Uveal melanocytic proliferation

**DOI:** 10.1186/s12886-020-01720-6

**Published:** 2020-12-04

**Authors:** Jingli Guo, Wenyi Tang, Wei Liu, Min Zhou, Qing Chang, Chunhui Jiang, Gezhi Xu, Wenji Wang, Qian Chen

**Affiliations:** 1 Department of Ophthalmology, Eye and ENT Hospital of Fudan University, Shanghai, 200031 China; 2 NHC Key Laboratory of myopia (Fudan University), Shanghai, China; 3 Shanghai Key Laboratory of Visual Impairment and Restoration, Shanghai, China

**Keywords:** Bilateral diffuse uveal melanocytic proliferation, Ultrasound biomicroscopy, High-frequency B-scan ultrasonography, Ciliary body nevi

## Abstract

**Background:**

To report undescribed characteristics of patients with bilateral diffuse uveal melanocytic proliferation (BDUMP) on ultrasound biomicroscopy (UBM) and high-frequency B-scan ultrasonography.

**Case presentation:**

Two of four participants presented with worsening bilateral vision after previously diagnosed primary pulmonary or ovarian carcinoma. The other two patients were diagnosed with lung carcinoma after presentation with BDUMP. All patients had ciliary body nevi-like lesion in combination with iris or ciliary body cysts, and uveal thickening on UBM. Focally elevated choroidal nevi-like lesion and exudative retinal detachment with choroidal thickening were detected with B-scan ultrasonography.

**Conclusions:**

Our case series demonstrates the uveal characteristics of patients with BDUMP based on high-frequency B-scan ultrasonography and UBM. Ultrasonographic findings are crucial in the diagnosis of BDUMP because it is occult in nature.

## Background

Bilateral diffuse uveal melanocytic proliferation (BDUMP) is an infrequent paraneoplastic ocular syndrome with remarkable vision loss. The characteristic manifestations were described by Gass and colleagues [1], including the presence of multiple orange-red lesions with early hyperfluorescence on fundus fluorescein angiography (FFA), scattered melanocytic tumors in a diffusely thickened uveal tract, exudative retinal detachment, and rapidly progressive cataract. As well as these five cardinal signs, few studies have paid attention to the abnormalities of the iris and ciliary body lesions because of its occult manifestations. Here, we report a case series of patients with BDUMP, characterized by ciliary body and choroidal nevi-like lesions in combination with iris or ciliary body cysts, and diffuse uveal thickening detected with ultrasound biomicroscopy (UBM) and high-frequency B-scan ultrasonography in order to provide more theoretical basis for clinical diagnosis.

## Case presentation

### Case 1

A woman in her 60s presented with blurred vision and photophobia in both eyes, which had persisted for 3 months. She had previously been diagnosed with clear cell adenocarcinoma of the ovary in 2003 and was treated with surgical resection, with a total hysterosalpingo-oophorectomy, and chemotherapy administered as eight cycles of taxol and carboplatin. A gastric stromal tumor was then detected and surgically resected in 2012, and metastatic lymph nodes in the groin were detected in 2018. Her history of ocular surgery included cataract phacoemulsification and intraocular lens implantation for rapidly progressive cataract in 2018.

**Fig. 1:**
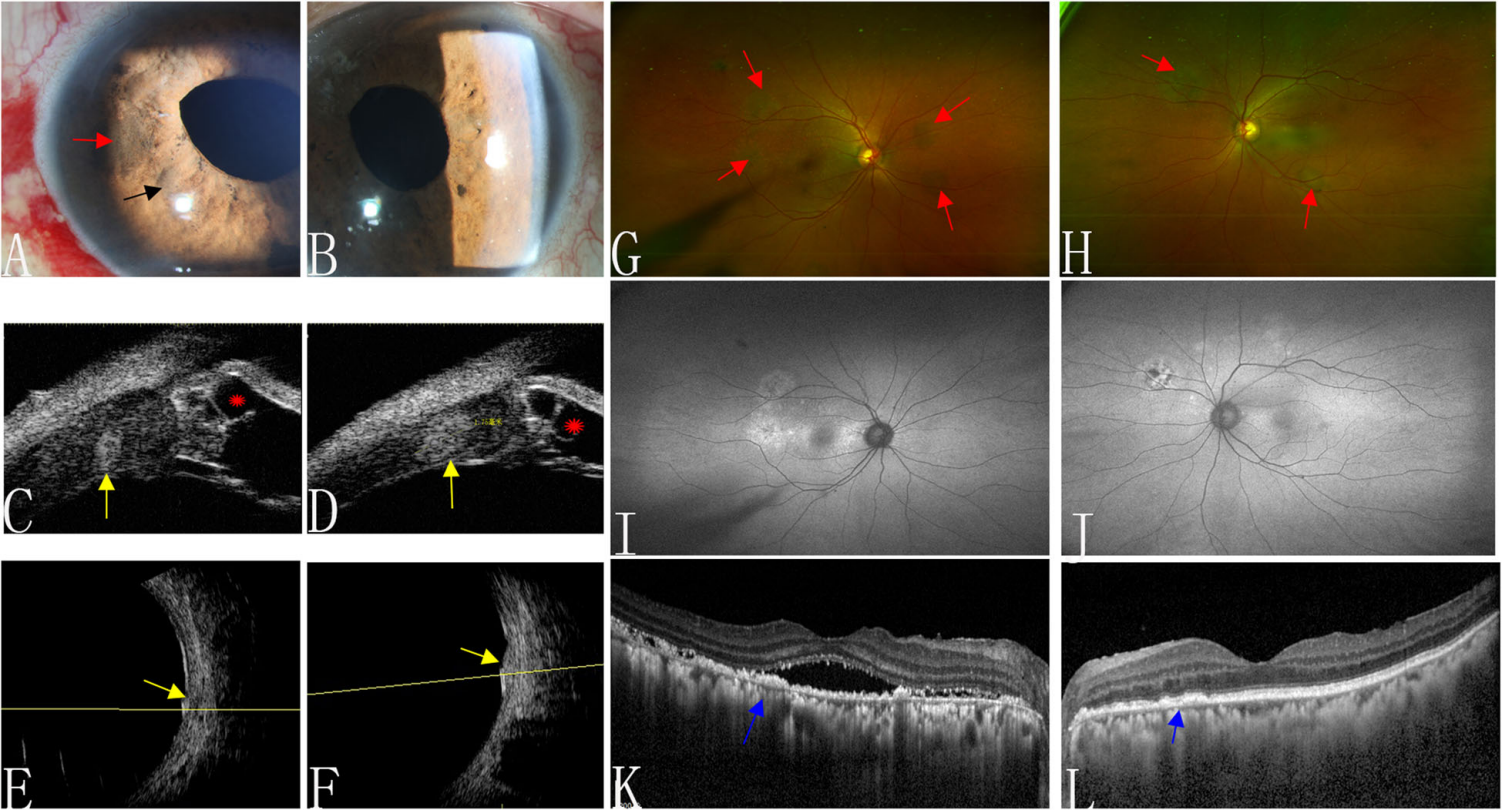
Multimodal imaging of Case 1. Anterior segment **a** and **b** showed iris nevus-like lesions (black arrow) and iris pigmentation (red arrow). Ultrasound biomicroscopy showed peripherally shallow anterior chamber, diffuse ciliary body thickening, a ciliary body nevi (yellow arrows), and ciliary body cysts (red asterisks) **c** and **d**. Bilateral diffuse choroidal thickening and a choroidal nevi (yellow arrows) were detected with high-frequency B-scan ultrasonography **e** and **f**. **g** and **h** show multiple, irregular, elevated, pigmented lesions (red arrows) and the corresponding hypoautofluorescence of these lesions on fundus autofluorescence **i** and **j** in the right and left eyes, respectively. Optical coherence tomography detected hyperreflective spots (blue arrows) in both eyes and subretinal fluid in the right eye

On presentation, the patient’s best-corrected visual acuity (BCVA) was 20/32 in the right eye and 20/25 in the left eye. An ophthalmoscopic examination revealed a peripherally shallow anterior chamber, with iris pigmentation and an iris nodule in the anterior chamber in both eyes (Fig. 1a and b). The intraocular pressure was 25/26 mmHg in both eyes. A fundus examination showed multiple, irregularly shaped, elevated pigmented lesions in the peripheral retina (Fig. 1g and h), corresponding to multifocal early nummular areas of hyperfluorescence on FFA and reduced autofluorescence (AF) (Fig. 1i and j). Optical coherence tomography (OCT) showed subretinal fluid in the right eye and irregular hyperreflective spots in both eyes (Fig. 1k and l). High-frequency B-scan ultrasonography revealed bilateral diffuse choroidal thickening and scattered choroidal nevi-like lesions (Fig. 1e and f). UBM showed a peripherally shallow anterior chamber, diffuse ciliary body thickening, ciliary body nevi-like lesions, and multiple ciliary body cysts (Fig. 1c and d).

### Case 2

**Fig. 2:**
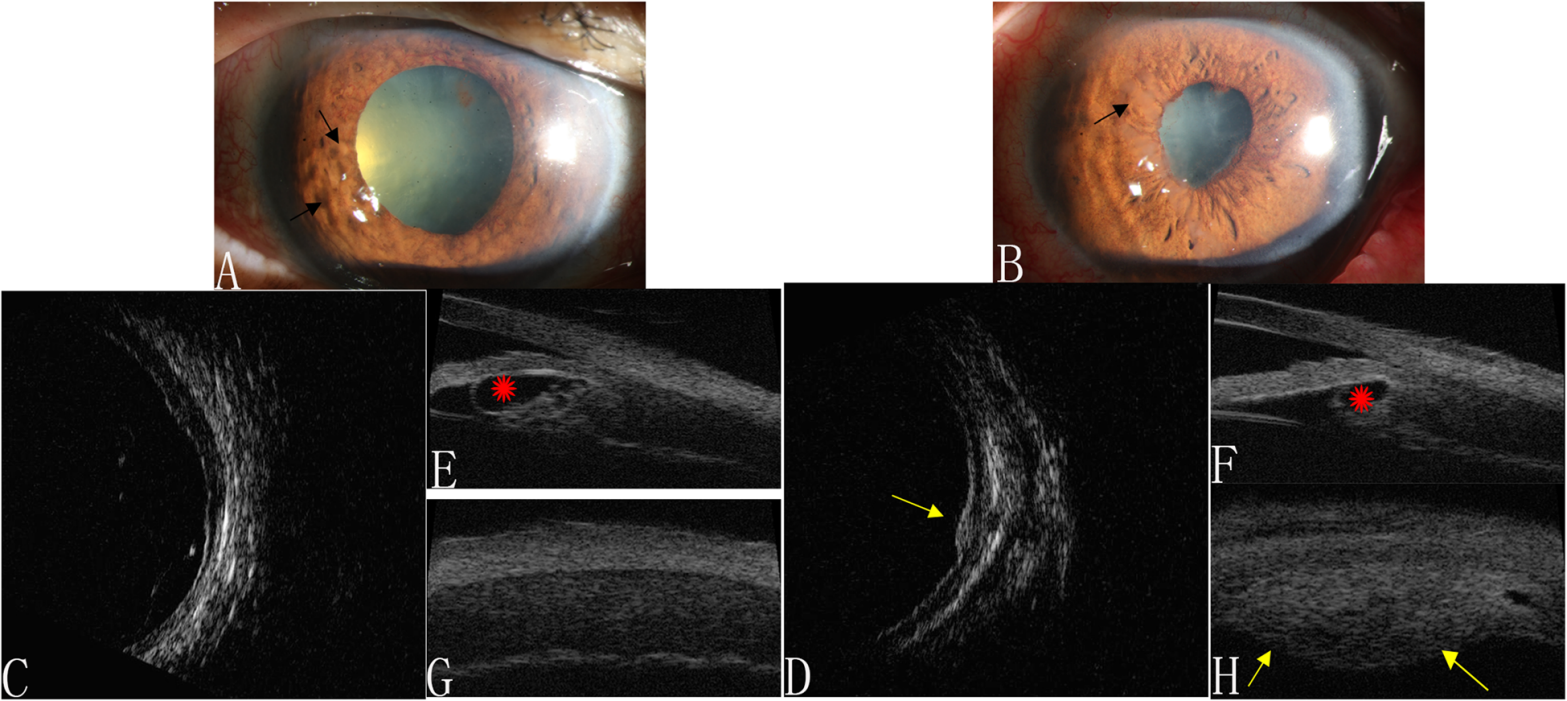
Presentation of Case 2. **a** slit-lamp examination revealed multiple iris nevus-like lesions (black arrows) and iris neovascularization in both eyes **a** and **b**. High-frequency B-scan ultrasonography of both eyes **c** and **d** revealed diffuse choroidal thickening and a choroidal nevi (yellow arrow). Iris cysts (red asterisks), a ciliary body nevi (yellow arrows), and diffuse ciliary body thickening were detected with ultrasound biomicroscopy

A 67-year-old man with a previous diagnosis of lung carcinoma presented with an 8-week history of worsening bilateral vision. On ophthalmological examination, his BCVA was finger counting in both eyes. Mild flare, iris neovascularization, and nevus-like lesions were present in both eyes (Fig. 2a and b). A slit-lamp examination revealed moderate bilateral cataracts in both eyes. A dilated fundus examination showed scattered pigmented choroidal lesions with exudative macular detachment in the right eye. Intervening areas of increasing and decreasing AF were observed. High-frequency B-scan ultrasonography revealed diffuse bilateral choroidal thickening and a choroidal nevi-like lesion (Fig. 2c and d). UBM confirmed shallow bilateral detachment of the ciliary body with diffuse ciliary body thickening, iridocyclitic cysts, and a ciliary body nevi-like lesion (Figs. 2e–h).

### Case 3

**Fig. 3:**
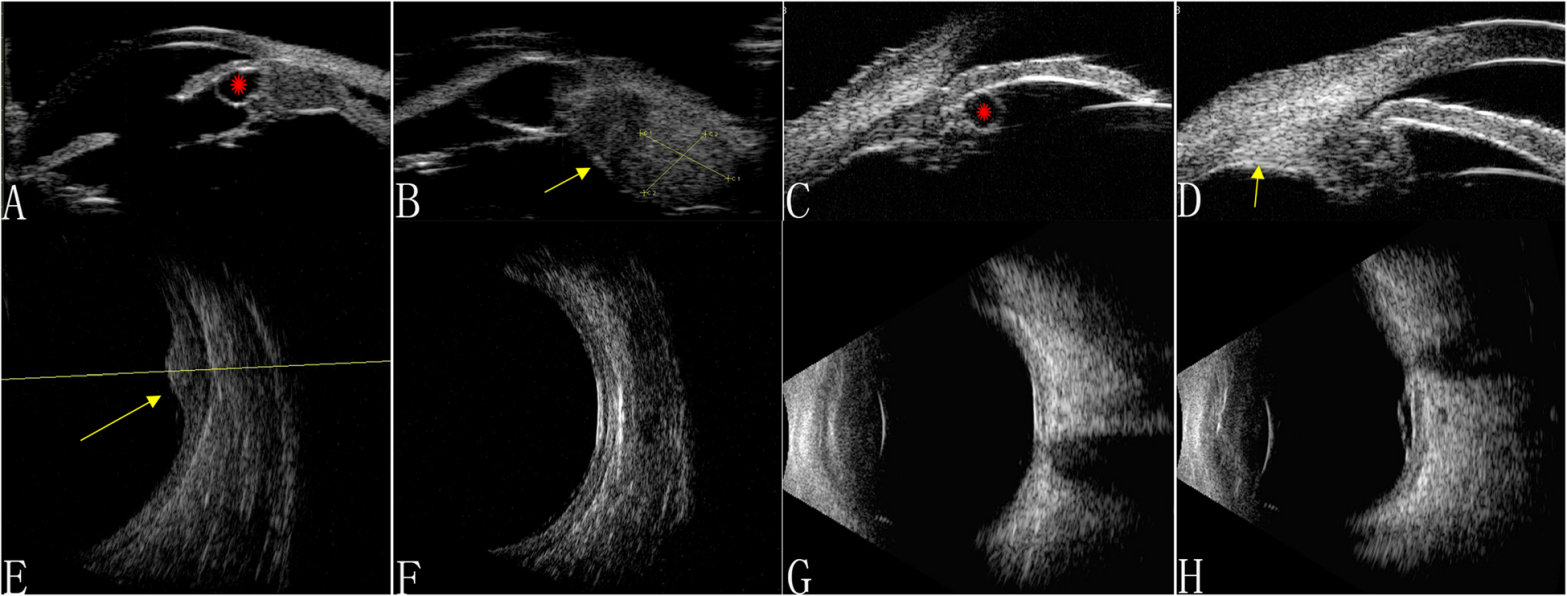
Ultrasonography findings of Case 3 and Case 4. Ultrasound biomicroscopy revealed multiple iris cysts (red asterisks), a ciliary body nevi (yellow arrow), and diffuse ciliary body thickening in Case 3 **a** and **b**) and Case 4 **c** and **d**. B-scan ultrasonography showed choroidal thickening and choroidal nevi in Case 3 **e** and **f** and Case 4 **g** and **h**

A 68-year-old Chinese man was referred with worsening bilateral vision, accompanied by weight loss and weakness over several months. Two years earlier, the patient had undergone phacoemulsification for rapidly progressive reduced vision. His visual acuity was 20/200 in both eyes. An anterior segment examination was remarkable for keratic precipitates in the corneal endothelium, mild flare in the anterior chamber, iris bumps, posterior synechia of the iris, and posterior capsule opacification. A funduscopic examination was invisible. A hyperecho choroidal nevi with diffuse choroidal thickening and focal exudative retinal detachment in the posterior fundus were detected in both eyes with high-frequency B-scan ultrasonography (Fig. 3e and f). UBM showed shallow ciliary detachment with diffuse ciliary thickening and multiple unequally sized ciliary cysts in both eyes, and a ciliary body nevi-like lesion in the left eye (Fig. 3a and b). Following a normal systemic workup, including computed tomography and a positron emission tomography scan, the man died from metastatic lung cancer.

### Case 4

A 67-year-old man complained of blurred bilateral vision complicated with headache lasting 20 days, with previously diagnosed uveitis that had been treated with intravenous methylprednisolone (200 mg/day) for 2 days, followed by oral prednisone (30 mg/day) and cyclosporine A (3 mg/kg/day). His BCVA was 20/25 in the right eye and 20/200 in the left eye. The anterior segments and intraocular pressure were normal in both eyes. Ophthalmoscopy showed multiple red patches at the level of the retinal pigment epithelium surrounding the optic disc, corresponding to early hyperfluorescence on FFA and reduced AF. OCT detected subretinal fluid in the bilateral macula. A solitary choroidal nevi-like lesion with choroidal thickening of the posterior pole was detected with B-scan ultrasonography (Fig. 3g and h). UBM confirmed a ciliary body cyst and a ciliary body nevi-like lesion (Fig. 3c and d). A systemic examination and immunohistochemical profile returned a diagnosis of primary pulmonary adenocarcinoma.

All these four patients were diagnosed with BDUMP. Demographic and clinical data of patinets with BDUMP were presented in Table 1. Their common specific characteristics on ultrasound included uveal nevi in combination with diffuse uveal thickening and uveal cysts, especially on high-resolution UBM, which revealed ciliary body nevi-like lesion in combination with iris or ciliary cysts and diffuse ciliary body thickening.

**Table 1:** Demographic and Clinical Data of Patients with Bilateral Diffuse Uveal Melanocytic Proliferation

		Case 1		Case 2		Case 3		Case 4	
Age/Sex/Race		60y/F/Asian		67y/M/Asian		68y/M/Asian		67y/M/Asian	
		OD	OS	OD	OS	OD	OS	OD	OS
OCT	Mean SFCT (μm)	536	514	496	–	–	–	525	502
UBM	MD of lesion (mm)	1.32	1.35	5.02	–	3.72	3.89	2.27	1.23
	MT of lesion (mm)	1.53	1.25	1.57	–	4.19	4.01	0.86	1.04
HF B-scan ultrasonography	MD of lesion (mm)	5.11	5.13	5.52	–	5.82	7.2	2.71	2.68
	MT of lesion (mm)	1.07	0.98	1.02	–	1.62	5.2	0.57	0.84

*HF* high frequency, *OCT* optical coherence tomography, *UBM* ultrasound biomicroscopy, *MD* maximum diameter, *MT* maximum thickness, *SFCT* subfoveal choroidal thickness, *OD* right eye, *OS* left eye, *F* female, *M* man

## Discussion and conclusion

Although Gass et al. reported that the five cardinal findings in BDUMP patients are usually associated with systemic malignancy [2, 3], the present descriptions demonstrate several other peculiar characteristics: uveal nevi-like lesion, iris and ciliary cysts, and diffuse uveal thickening. A previous study reported a patient with clear cell adenocarcinoma of the endometrium and progressive iris nevi in both eyes [4]. In contrast, our cases are notable for their ciliary and choroidal nevi-like lesion in combination with iris or ciliary body cysts, and diffuse ciliary body thickening. Joseph et al. described a patient with iris and ciliarybody cysts and diffuse thickening of the ciliary body [2]. However, ciliary nevi-like lesion were also common in our UBM images. Although iris and ciliary body cysts and diffuse ciliary thickening have previously been reported in association with BDUMP, patients in our case series also displayed characteristic ciliary body and choroidal nevi-like lesions. Establishing the involvement of the anterior uvea with high-resolution UBM is particularly helpful in the diagnosis of BDUMP because it not only detects the thickness of the uveal tract and the small nevi in it, but also the angle closure secondary to iris and ciliary cysts. Lesions of the iris and ciliary body may also contribute to rapidly progressive cataracts.

The lack of diffuse uveal thickening in Case 4 is attributed to the administration of methylprednisolone and cyclosporine A for about 2 months before he presented. However, our study emphasizes the importance of the uveal changes in treatment-naïve BDUMP patients.

Our case series shows that BDUMP can present with subtle clinical signs and is often caused by an occult malignancy. Therefore, the diagnosis is often delayed or mistaken and involves further clinical investigation. UBM and high-frequency B-scan ultrasonography are of the utmost importance in the diagnosis of BDUMP. To our knowledge, this report is the first to describe the appearance of the ciliary body and choroidal nevi-like lesions in combination with iris or ciliary cysts, and diffuse uveal thickening associated with BDUMP by high-resolution, fast and convenient ultrasonographic examinations.

## Data Availability

All data analysed during the current study available from the corresponding author on reasonable request.

## Abbreviations

AF: Autofluorescence
BCVA: Best-corrected visual acuity
BDUMP: Bilateral diffuse uveal melanocytic proliferation
FFA: Fundus fluorescein angiography
OCT: Optical coherence tomography
UBM: Ultrasound biomicroscopy
